# *In vivo* assessment of inflammation in carotid atherosclerosis by noninvasive photoacoustic imaging

**DOI:** 10.7150/thno.41211

**Published:** 2020-03-26

**Authors:** Zhihua Xie, Yanqing Yang, Yaqiong He, Chengyou Shu, Dong Chen, Jinke Zhang, Jingqin Chen, Chengbo Liu, Zonghai Sheng, Huadong Liu, Jie Liu, Xiaojing Gong, Liang Song, Shaohong Dong

**Affiliations:** 1 Research Laboratory for Biomedical Optics and Molecular Imaging, Institute of Biomedical and Health Engineering, Shenzhen Institutes of Advanced Technology (SIAT), Chinese Academy of Sciences, 1068 Xueyuan Avenue, Shenzhen 518055, China.; 2 Key Laboratory of Flexible Electronics (KLOFE) Institute of Advanced Materials (IAM), Nanjing Tech University (Nianjing Tech), 30 South Puzhu Road, Nanjing 211800, China.; 3 Department of Cardiology, Shenzhen People's Hospital, the Second Affiliated Hospital, Jinan University, 1017 Dongmen North Road, Shenzhen 518020, China.; 4 CAS Key Laboratory of Health Informatics, Shenzhen Institutes of Advanced Technology, Chinese Academy of Sciences, 1068 Xueyuan Avenue, Shenzhen 518055, China.

**Keywords:** molecular imaging, PBD-CD36, noninvasive photoacoustic imaging, carotid atherosclerosis, inflammation

## Abstract

**Objectives**: The objective of this study was to demonstrate the feasibility of using noninvasive photoacoustic imaging technology along with novel semiconducting polymer nanoparticles for *in vivo* identifying inflammatory components in carotid atherosclerosis and assessing the severity of inflammation using mouse models.

**Methods and Results**: Healthy carotid arteries and atherosclerotic carotid arteries were imaged *in vivo* by the noninvasive photoacoustic imaging system. Molecular probes PBD-CD36 were used to label the inflammatory cells to show the inflammation information by photoacoustic imaging. In *in vivo* imaging experiments, we observed the maximum photoacoustic signal enhancement of 4.3, 5.2, 8 and 16.3 times between 24 h post probe injection and that before probe injection in four carotid arteries belonging to three atherosclerotic mice models. In the corresponding carotid arteries stained with CD36, the ratio of 0.043, 0.061, 0.082 and 0.113 was found between CD36 positive (CD36(+)) expression area and intima-media area (P < 0.05). For the CD36(+) expression less than 0.008 in eight arteries, no photoacoustic signal enhancement was found due to the limited system sensitivity. The photoacoustic signal reflects CD36(+) expression in plaques, which shows the feasibility of using photoacoustic imaging for* in vivo* assessment of carotid atherosclerosis.

**Conclusion**: This research demonstrates a semiconducting polymer nanoparticle along with photoacoustic technology for noninvasive imaging and assessment of inflammation of carotid atherosclerotic plaques *in vivo.*

## Introduction

The vascular system dysfunction due to atherosclerosis is the leading cause of severe health, mortality, and economic problems around the world. The plaque buildup in the walls of carotid arteries subsequently leads to luminal stenosis, low blood supply to the brain, and even acute stroke incidents with plaque rupture 1,2. Inflammation, which occurs at plaque locations silently for decades before plaque formation, is widely considered as a prominent factor in atherosclerosis progression and plaque vulnerability 3-5. Detecting inflammation activities enables early diagnosis and accurate risk assessment of atherosclerosis. Molecular imaging has been explored with great specificity to detect inflammation by targeting certain inflammatory compositions, like macrophages, myeloid cells and vascular cell adhesion molecule-1 (VCAM-1) 6-10. Some popular molecular imaging modalities to understand the atherosclerosis progression mechanism are computed tomography (CT) 11, positron emission tomography (PET) 6,7, and magnetic resonance imaging (MRI) 8. These technologies are either expensive or harmful due to radiation, limiting their use for routine clinical evaluation of atherosclerosis. The radiation-free and low-cost imaging modalities based on light or ultrasound have also been explored in clinical and experimental studies. Molecular fluorescence imaging is an optical modality for visualizing atherosclerotic plaque inflammation with high sensitivity and excellent imaging resolution 12-15. This modality only offers two-dimensional (2D) signal distributions and lacks signal depth resolution. In addition, it's difficult to achieve good resolution in biological tissue beyond 1 mm due to light scattering in the medium 15. Ultrasound (US) imaging is a commonly-used clinical imaging technology to provide accurate three-dimensional (3D) structural information of tissues. The application of this modality in atherosclerotic inflammation detection is limited by the penetration ability of micron-sized contrast agents 9,10,16. Although nano bubbles are developed to improve their penetration to vasculature, bubble size contradicts its stability and echogenicity efficiency in clinical US systems 17-19. Therefore, there is a need that exists for a new imaging modality which is radiation-free, low cost, has various nano-sized contrast agents and provides accurate 3D imaging information of atherosclerotic inflammation with high contrast.

The photoacoustic (PA) modality is an emerging imaging technology that combines the advantages of optics and ultrasound modalities, with the potential to detect atherosclerotic inflammation 20. In PA imaging, targets absorb diffused photons and release PA signal. The imaging resolution and imaging depth are comparable to that of US imaging. Meanwhile, the imaging contrast and sensitivity are as high as that of optical fluorescence imaging, enabling accurate 3D imaging in deep tissues 21-23. For* in vivo* PA imaging, the excitation light at visible range or near infrared I (NIR I: 650-900 nm) suffers from strong scattering in biological tissues, while the light in NIR II (NIRII: 1000-1700 nm) exhibits reduced light scattering 24,25. The reduced scattering improves PA imaging depth and spatial resolution 26-28. Noninvasive PA imaging modality has been used to obtain the information of carotid atherosclerosis inflammation or calcification* ex vivo* 29, 30. Professor Vasilis's group applied PA technology to healthy human carotid artery imaging *in vivo* with blood as PA signal source 31. *In vivo* carotid artery thrombosis imaging based on mouse models has been reported 32. The previous studies demonstrated the potential ability of noninvasive PA imaging for carotid atherosclerosis inflammation detection *in vivo*.

To detect carotid atherosclerosis inflammation efficiently *in vivo* with noninvasive PA imaging, developing PA molecular probes loaded with specific labeling marker and having the absorbance at NIRII range is essential. So far, several semiconducting polymer nanoparticles (NPs) have been proposed with tunable optical properties, controllable dimensions, high extinction coefficient, excellent photostability and biocompatibility 33,34. They generally exhibit much better PA properties than other types of PA contrast agents, thus they have become a “hotspot” in biomedical area. Very recently, we have demonstrated that semiconducting polymer NPs with NIR-II region (1000-1400 nm) for PA imaging afforded deeper tissue penetration and higher signal-to-noise ratio compared to that in the traditional NIR region (650-900 nm) 35, due to reduced light scattering and reduced background. Until now, semiconducting polymer NPs have mainly been applied in tumor diagnosis and therapy 36-39, osteomyelitis diagnosis and therapy 40. However, none of them has been applied in *in vivo* noninvasive assessment of inflammation in carotid atherosclerosis. In this study, we will develop a semiconducting polymer NP for atherosclerosis inflammation labeling. To label atherosclerotic inflammatory cell efficiently, CD36 is considered as an important marker. The CD36 participates in atherosclerotic arterial lesion formation through the uptake of oxidized low-density lipoprotein (oxLDL) and the trigger of signaling cascades for inflammatory responses. Thus it could be used to study the disease progression as an effective marker 41.

In this research, an anti-CD36 decorated semiconducting polymer NP (PBD-CD36 NP) with the absorption wavelength of 1064 nm are developed for *in vivo* imaging of atherosclerotic inflammation in the carotid arteries of mouse. We validate PA imaging results in mouse models with and without atherosclerotic plaques and under different inflammation severity using immunohistochemistry staining. This study is a comprehensive work that comprises noninvasive PA imaging technology, semiconducting polymer NPs at NIR II range with efficient target, with the mouse carotid atherosclerosis models qualified for statistical analysis. The work demonstrated the feasibility and potential of noninvasive PA molecular imaging technology in *in vivo* identifying and assessing carotid atherosclerosis inflammation for the first time, to the best of our knowledge. Our study contribution is significant towards advancing the use of PA technology for atherosclerosis imaging, thus potentially benefiting patients who have atherosclerosis. In the next few sections, we present the principals of our study in detail, along with the results and discussion.

## Material and Methods

### Materials

1,2-Distearoyl-*sn*-glycero-3-phosphoetanolamine-*N*-[methoxy(polyethylene glycol)-2000] (DSPE-PEG_2000_) and 1,2-distearoyl-*sn*-glycero-3-phosphoetanolamine-*N*-[maleimide(polyethylene glycol)-2000] (DSPE-PEG_2000_-Mal) were obtained from Laysan Bio, Inc. Anti-CD36 was purchased from Abcam. Milli-Q water (18.2 MΩ) was supplied by a Milli-Q Plus System (Millipore Corporation, Bedford, USA) and used for all the experiments requiring aqueous medium. 4,8-Dibromo-6-(2-ethylhexyl)-[1,2,5]thiadiazole[3,4-*f*]benzotriazole and (4,8-bis((2-octyldodecyl)oxy)benzo[1,2-*b*:4,5-*b'*]dithiophene-2,6-diyl)bis(trimethylstannane) were purchased from Derthon Optoelectronic Materials Science Technology Co LTD. All other chemicals were obtained from Sigma-Aldrich or Energy Chemical (China) and used without further purification.

### Characterization

The hydrodynamic diameter and zeta potential of PBD-CD36 NPs were recorded on Micromeritics Nanoplus-3. Transmission electron microscopy (TEM) images were measured on a JEOL JEM-2100 electron microscope with an accelerating voltage of 200 kV. UV-vis-NIR spectra were measured on a Shimadzu UV-1750 spectrometer.

### Preparation of PBD

A mixture of (4,8-bis((2-octyldodecyl)oxy)benzo[1,2-*b*:4,5-*b'*]dithiophene-2,6-diyl)bis(trimethylstannane) (110.8 mg, 0.1 mmol), 4,8-dibromo-6-(2-ethylhexyl)-[1,2,5]thiadiazolo[3,4-*f*]benzotriazole (44.7 mg, 0.1 mmol), Pd2_(dba)3_ (5.0 mg, 5.4 µmol) and P(*o*-tolyl)_3_ (5.0 mg, 16.2 µmol) in toluene (10 mL) was stirred at 100 °C for 24 h under argon atmosphere. After cooling down to room temperature, the mixture was dropped slowly into methanol (100 mL) to precipitate the crude polymer followed by centrifugation. The crude polymer was subsequently re-dissolved in dichloromethane (200 mL), washed with water for 3 times, and dried over MgSO_4_. After filtration with filter paper, the organic phase was concentrated to ~10 mL, which was added slowly to methanol (100 mL). Then PBD (71 mg, yield: 67%) was collected as a black solid by centrifugation.

### Preparation of PBD NPs and PBD-CD36 NPs

A mixture of PBD (1 mg) and DSPE-PEG_2000_ (2 mg) dissolved well in THF (1 mL) was injected into water (10 mL), which was followed by sonication for two minutes at 195 W output using a microtip probe sonicator. The mixture was then stirred overnight in fumehood at room temperature to evaporate THF. After filtration with a 0.2 µm syringe drive filter, the obtained NPs were concentrated to be 1 mg/mL using an ultrafilter (molecular weight cut off = 30 K), and stored at 4 °C for further use.

For the NPs with maleimide functional groups on the surface, the procedure was similar only by replacing 10% of DSPE-PEG_2000_ with the same mass of DSPE-PEG_2000_-Mal. The following conjugation reaction was performed *via* click reaction. In brief, prior to conjugation with the maleimide functionalized NPs, anti-CD36 (20 μg) was treated with 2, 4, 5-trichlorophenoxyethanol (TCPE) (5 mg) for 30 min at room temperature. Then the resultant mixture and maleimide-functionalized NPs (1 mg) were stirred in 20 mM PBS solution for 24 h. After dialysis against with water (molecular weight cut off = 100 K), the solution was filtered with a 0.2 µm syringe drive filter, the obtained NPs were concentrated to be 1 mg/mL using an ultrafilter (molecular weight cut off = 30 K). The synthetic routes of PBD NPs and PBD-CD36 NPs are shown in Figure 2A-B.

### Animal Preparation

All the procedures were performed according to a protocol approved by the Animal Study Committee of Shenzhen Institute of Advanced Technology, Chinese Academy of Sciences. Fifteen male ApoE-/- mice (8-week-old, 15 g weight) were purchased from Model Animal Research Center of Nanjing University, China. Ten mice were continually fed with a high-fat diet (HFD) (containing 15% fat and 1.25% cholesterol) for 16 weeks to induce atherosclerosis. Five mice belonging to the control group were fed with a normal diet. The PA imaging of the mice was performed 17 weeks post diet initiation.

### Blood circulation

PBD-CD36 NPs (1 mg/mL, 150 μL) were injected via tail vein into three healthy mice. About 20 µL blood samples were taken from the tail end at 0 h, 0.5 h, 1 h, 3 h, 5 h, 7 h, 10 h, 23 h, and 25 h post probe injection and removed to plastic tubes. The blood samples in plastic tubes were imaged immediately using the PA imaging system. The probe concentration in blood was quantified by averaging the PA signals of the blood samples.

### *In vivo* photoacoustic imaging operations

**Figure 1:**
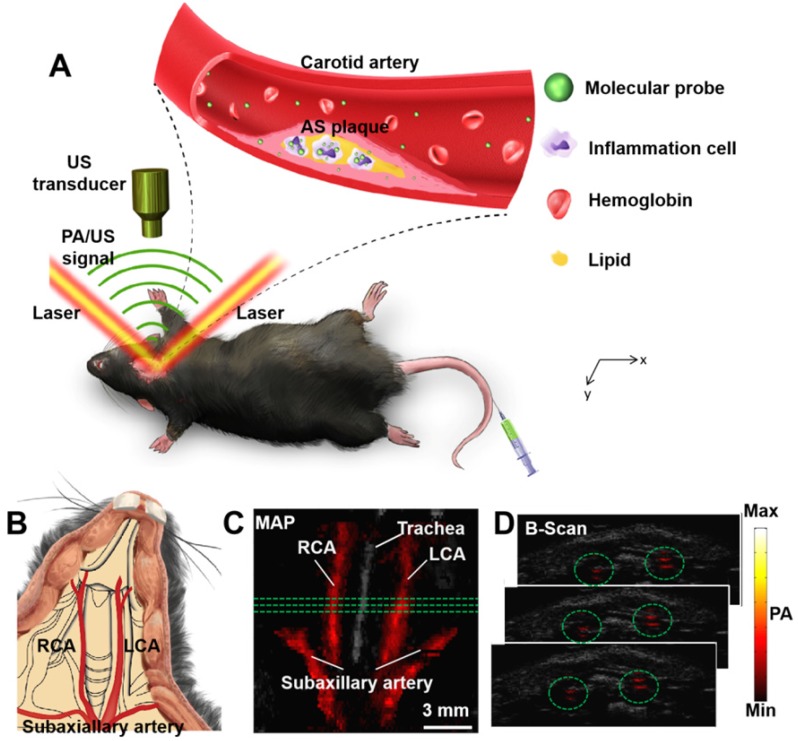
** (A)** Principle of carotid atherosclerotic inflammation detection. A beam of excitation light is focused onto the shaved mouse neck, generating a PA signal which is detected by an ultrasonic transducer. **(B)** Schematic anatomy of carotid arteries of mice. PA/US fused MAP (maximum amplitude projection) image **(C)**; and consecutive B-scan images **(D)**. The PA signal in green circles in (C) is from the carotid arteries at the three green lines in (B). Ultrasound image: gray; Photoacoustic image: hot. AS plaque: atherosclerotic plaque; LCA: Left Carotid Artery; PA: photoacoustic; RCA: Right Carotid Artery; US: ultrasound.

All the *in vivo* PA imaging experiments in this research were based on a custom-built PA/US imaging system which have been described in previous studies 42. A laser (Innolas Spitlight 600, OPO) with repetition rate of 20 Hz was used as excitation source in this research. The excitation wavelength is at 1064 nm. The optical fluence of the excitation laser focused on the animal surface which was approximately 12 mJ/cm^2^ (below the ANSI safety threshold). Scanning area was fixed to 14 mm × 10 mm, with point-to-point (A-line) scanning step of 0.1 mm and frame-to-frame (B-scan) scanning step of 0.2 mm (140 A-lines/B-scan, 50 total B-scans). To acquire one volumetric image, it took approximately 8.5 min. During the experiment, the mice were scanned and imaged at the shaved neck area (Figure 1B). The mice were placed on a heating plate (37 °C) and were fully anesthetized using isoflurane (1.5% isoflurane gas mixed with oxygen). The principal of PA imaging is shown in Figure 1A and the PA images of carotid arteries *in vivo* are shown in Figure 1C-D. 3D information of the carotid arteries can be obtained in PA images (Figure 1C-D).

### *In vivo* photoacoustic imaging of carotid arteries with and without atherosclerotic plaque

PBD NPs or PBD-CD36 NPs (1 mg/mL, 150 μL) were injected systematically into mice via tail vein. PA images of atherosclerotic models and control mice were acquired before and up to 24 h post NPs injection. The 24 h imaging time point was chosen to make sure NPs thorough elimination from the blood.

### Quantification of PA signal enhancement

The PA signal enhancement is quantified according to the following steps:

- On the PA images 24 h post NPs injection, we choose the volumetric region of interest which is composed of three or four consecutive B-scans; then determine the corresponding region of interest from the PA images obtained before NPs injection.
- The PA signal of a target artery from one B-scan is quantified by summing the PA intensity of all the pixels from the artery on the B-scan.
- The PA signal of the region of interest is quantified by averaging the PA signals of the chosen B-scans in the region.
- The PA signal enhancement is quantified by calculating the ratio of the quantified PA signal 24 h post NPs injection to that before NPs injection. An example of PA signal enhancement quantification can be found in supplementary material (Figure S7).

### Immunohistochemistry staining

Immunohistochemistry staining was performed to verify the *in vivo* imaging results. The carotid arteries with and without atherosclerotic plaques were excised and fixed in 4% paraformaldehyde for two days. They were subsequently embedded in optimal cutting temperature (OCT) compound and were sectioned for 4 μm thickness. Sections of each group of carotid arteries were incubated with rabbit anti-CD36 antibodies (1:100 dilution, Abcam 80080) at 37 °C for 30 min, and were left overnight at 4 °C. The eight sections of each sample were analyzed by immunohistochemistry staining, and stained images were captured by the microscope (Dmi8 + DFC 7000T Leica).

## Results

### Characterization of PBD-CD36 NPs

**Figure 2:**
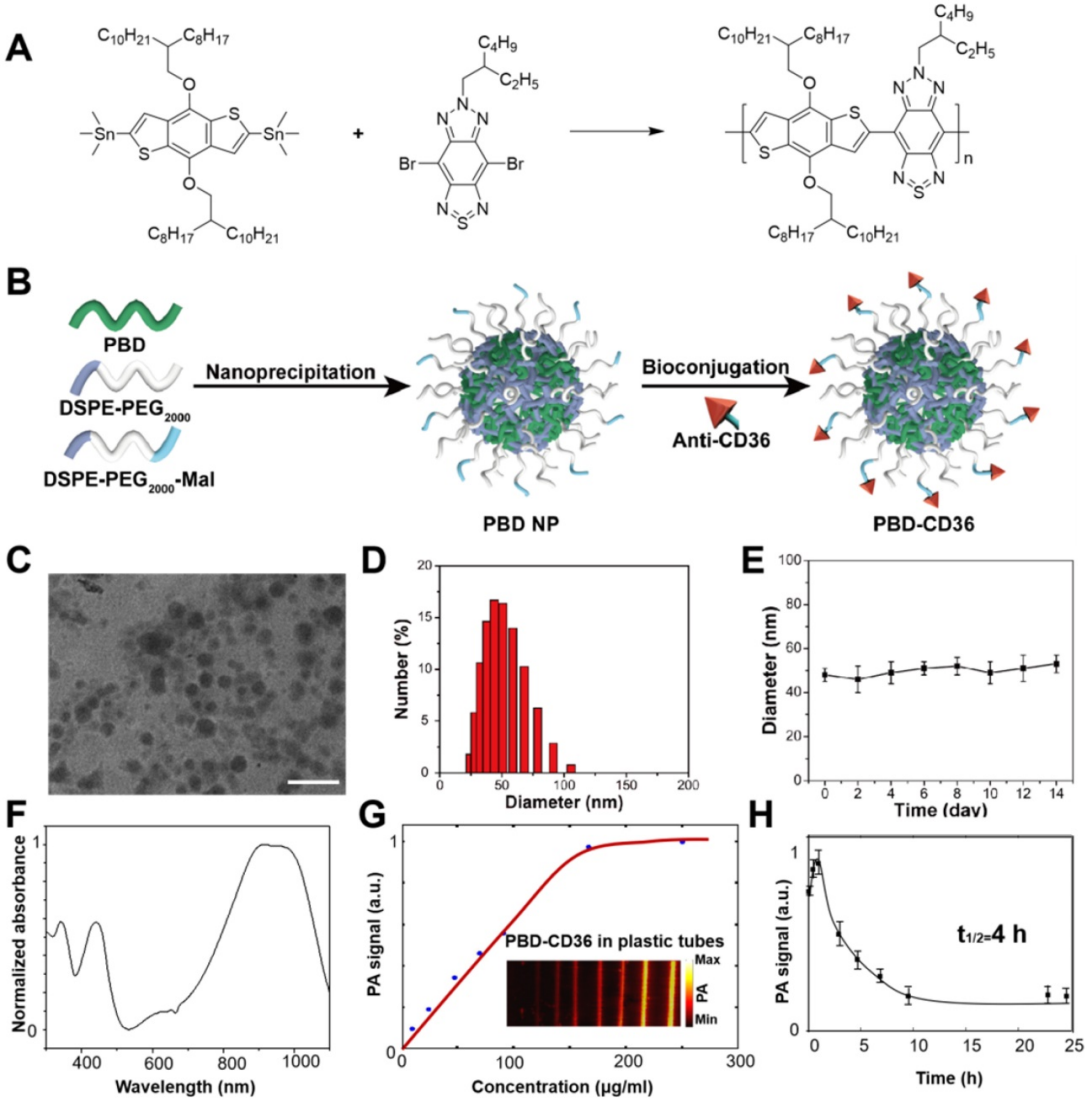
** (A)** Synthetic route to PBD; **(B)** Schematic illustration of the preparation of PBD-CD36; **(C)** TEM image of PBD-CD36. The scale bar represents 200 nm; **(D)** NP size distribution. **(E)** Stability study of PBD-CD36 in water over 14 days. Bars show means ± standard deviation (n = 3). **(F)** Absorption spectrum of the PBD-CD36 NPs. **(G)** PA signal of PBD-CD36 at different concentration with the excitation wavelength of 1064 nm. **(H)** Circulation curve of PBD-CD36 NPs in the blood of ApoE-/- mouse, n = 3.

Transmission electron microscopy (TEM) demonstrates the main diameter of PBD-CD36 at about 50 nm, and illustrates the spherical morphology and homogeneous particle size distribution (Figure 2C,D). The results of ^1^H NMR spectrum of PBD in CDCl_3_ and Zeta potential of PBD NPs and PBD-CD36 NPs are shown in supplementary materials (Figure S1 and S2). Stability study of PBD-CD36 in water is shown in Figure 2E, no obvious size variation following storage over 14 days, indicating the high colloidal stability of the NPs. The stabilities of PBD-CD36 NPs in different media are shown in Figure S3 and S4.

The absorption spectrum of the NP is shown in Figure 2F. The high absorption spectrum ranges from 850 nm to 1100 nm. For less scattering in tissues at longer wavelength, we choose 1064 nm as excitation wavelength in this research. The PA signal of the NPs at 1064 nm increases with increased concentrations and reaches saturation at a concentration of 160 μg/mL, as shown in Figure 2G. PBD-CD36 NPs circulation time in ApoE-/- mice is about 4 h as shown in Figure 2H and at the time point of 24 h, the negligible NPs are left in circulating blood.

### Toxicity study of PBD-CD36

**Figure 3:**
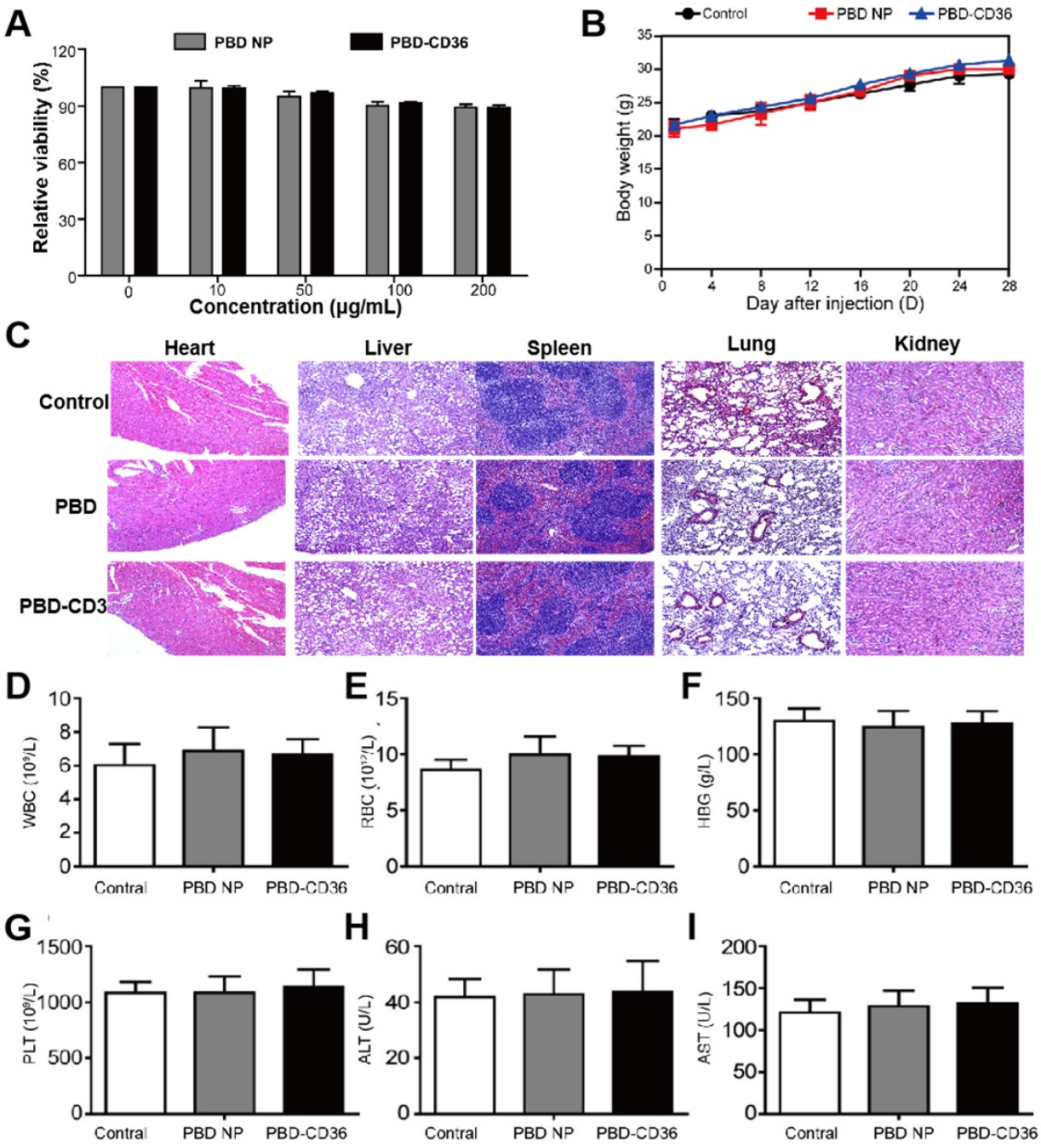
** (A)** Metabolic viability of Raw246.7 cells after incubation with PBD NPs and PBD-CD36 at different concentrations for 24 h. **(B)** Body weight measurements of mice treated with PBS, PBD NPs and PBD-CD36. Bars show means ± SD; n = 3. **(C)** Pathological images (H&E staining) of key organs including liver, spleen, kidney, heart and lung. (D)-(I) Blood index measurement of mice treated with PBS (control), PBD NPs and PBD-CD36. **(D)** WBC: white blood cells; **(E)** RBC: red blood cells; **(F)** HGB: hemoglobin; **(G)** PLT: platelets; **(H)** ALT: alanine aminotransferase; **(I)** AST: aspartate aminotransferase.

Raw246.7 cells incubate with PBD NPs and PBD-CD36 at different concentrations for 24 h. The result in Figure 3A (Figure S5) demonstrates good metabolic viability of cells in the NPs. Results in Figure 3B show no obvious change in the body weight of mice treated with PBS, PBD NPs and PBD-CD36. Pathological images (H&E staining) of key organs including liver, spleen, kidney, heart and lung of the mice shows negligible damage from the NPs (Figure 3C). Blood index measurement of mice treated with PBS (control), PBD NPs and PBD-CD36 was performed. The results revealed that white blood cells (WBC) (Figure 3D), red blood cells (RBC) (Figure 3E), hemoglobin (HGB) (Figure 3F), platelets (PLT) (Figure 3G), alanine aminotransferase (ALT) (Figure 3H) and aspartate aminotransferase (AST) (Figure 3I) were not significantly changed by PBD NPs and PBD-CD36 treatment for 7 and 28 days. The toxicity research of the NPs suggests their low toxicity under experimental conditions.

### *In vivo* photoacoustic imaging of atherosclerotic plaque with PBD and PBD-CD36 injection

**Figure 4:**
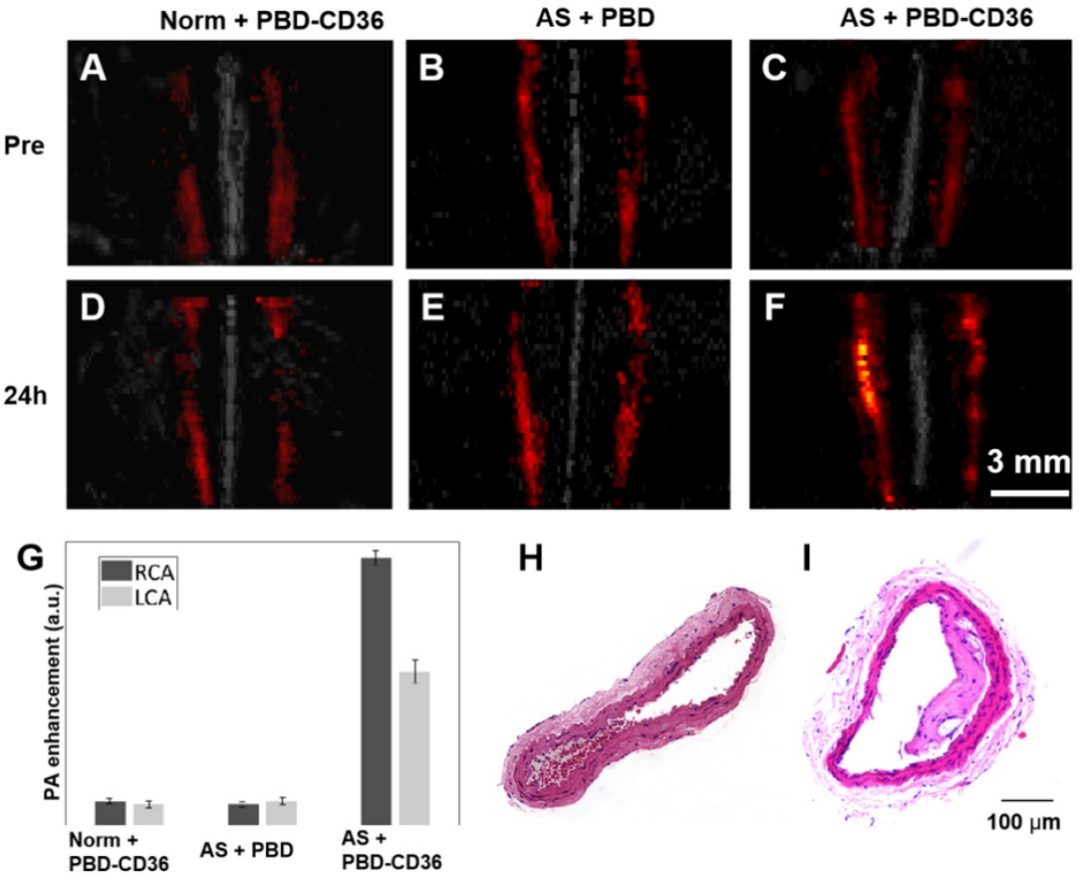
** (A-C)** Representative fused MAP images (PA/US image) of a normal mouse and two atherosclerostic mice, respectively. **(D-F)** Representative fused MAP image of normal mouse 24 h post PBD-CD36 NPs injection (D), atherosclerotic mouse 24 h post PBD NPs injection (E), and atherosclerotic mouse 24 h post PBD-CD36 NPs injecteion (F). **(G)** Quantified PA signal enhancement, n = 3. **(H-I)** The H&E stained carotid arteries of a normal mouse and atherosclerotic mouse, respectively.

The PA/US fused images of healthy mice with PBD-CD36 NPs injection (Norm + PBD-CD36), atherosclerotic mice with PBD NPs injection (AS + PBD) and atherosclerotic mice with PBD-CD36 NP injection (AS + PBD-CD36) are shown in Figure 4A-F. Representative H&E staining results of carotid arteries excised from normal mouse and atherosclerotic mouse in Figure 4H-I demonstrate obvious plaque formation in atherosclerotic mouse. In the imaging results of Figure 4A-F, we can find obvious PA signal enhancement for the group of AS + PBD-CD36 24 h post NPs injection, while the results of AS + PBD group and Norm + PBD-CD36 group shows no obvious signal enhancement post NPs injection, but with limited signal fluctuation (within 18%). The signal of the three groups shown in Figure 4G verified high PA signal enhancement at the group of AS + PBD-CD36.

### *In vivo* photoacoustic imaging of atherosclerotic plaque with different severity

**Figure 5:**
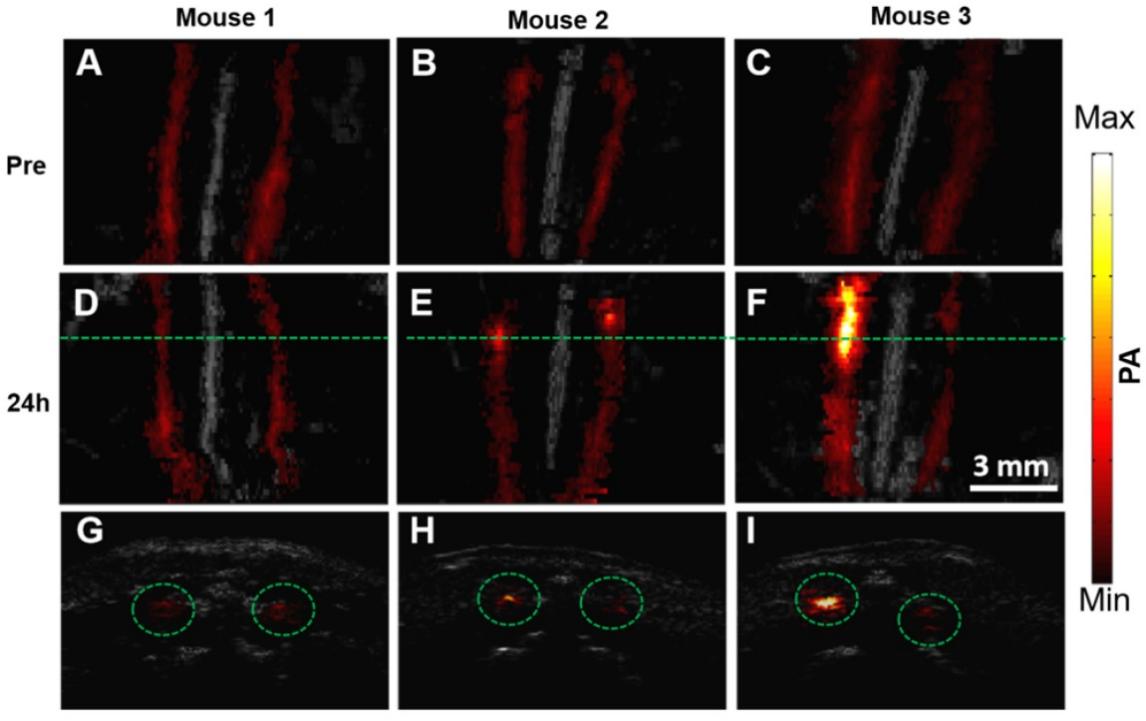
PA/US fused MAP images of three mice before **(A-C)** and 24 h post **(D-F)** molecular probes injection. **(G-I)** B-scan images at the green line locations in (D-F). The scale bar of the figure is shown in (F).

The representative PA/US fused images of healthy mice and two mouse models with different atherosclerosis severity, before and 24 h post PBD-CD36 injection are shown in Figure 5A-F, respectively. The control mouse on a normal diet (mouse 1) shows limited PA signal fluctuation (within 18%), but no obvious enhanced PA signal along with two arteries post probe injection turned up. The two atherosclerotic mouse models (mouse 2 and 3) with HFD show enhanced PA signal at a certain segment along the arteries, showing the existence of atherosclerotic plaques in these areas. The B-scan images along the green lines in Figure 5D-F are shown in Figure 5G-I to illustrate PA enhanced B-scan images.

### Immunohistological analysis of atherosclerosis inflammation severity

**Figure 6:**
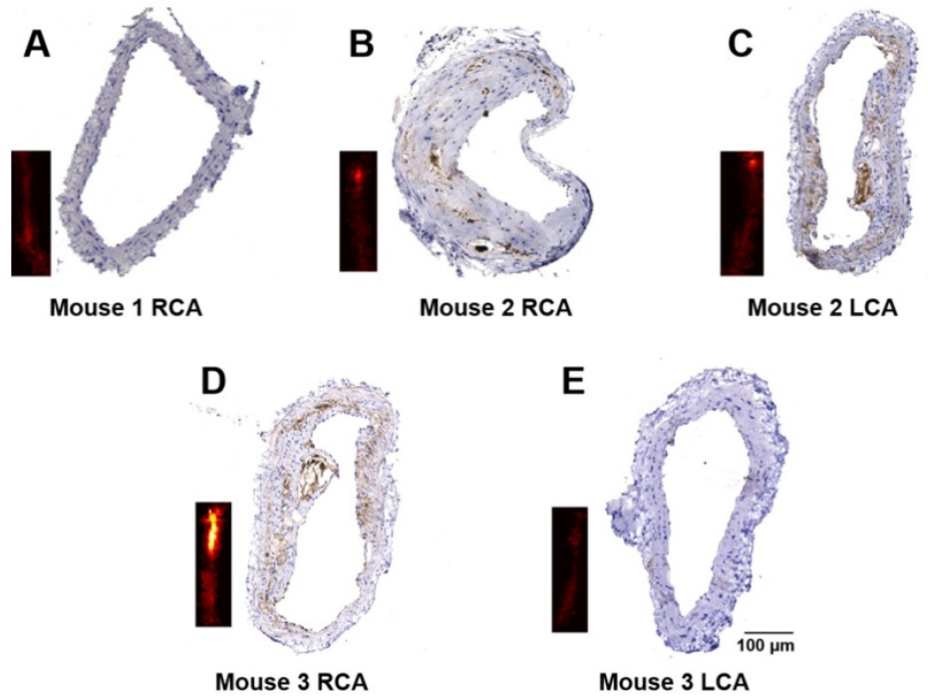
Immunohistochemistry staining of the carotid arteries. Representative results of CD36 staining of carotid arteries excised from a healthy mouse **(A)**; and atherosclerotic mice** (B-E)**. The insets are PA images of the corresponding carotid arteries.

Immunohistochemistry staining for CD36 was performed to verify the severity of atherosclerosis. The representative results of CD36(+) staining of the arteries in Figure 5 are shown in Figure 6. Obvious differences in expression of CD36(+) in different arteries can be seen. No CD36(+) expression and plaques are found in mouse 1 arteries and mouse 3 LCA (Figure 6A and Figure 6E), while different levels of CD36(+) expression were seen in mouse 2 RCA, LCA and mouse 3 RCA as shown in Figure 6B-D, respectively.

**Figure 7:**
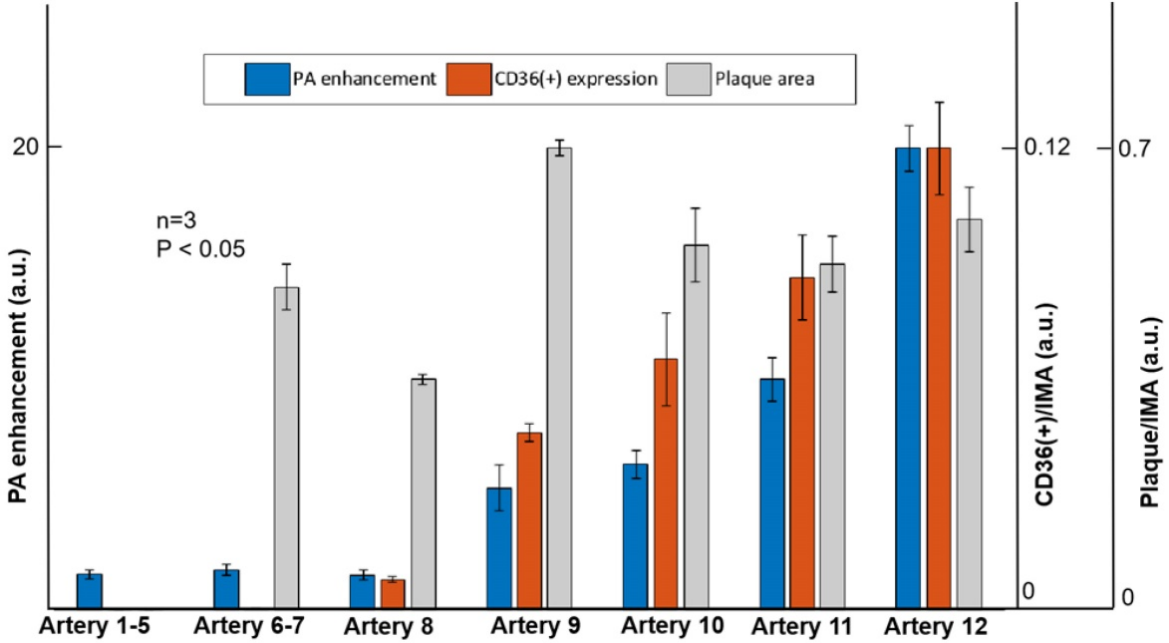
Quantified PA enhancement, the ratio of CD36 (+) expression area to intima-media area (IMA), and the ratio of plaque area to IMA of seven arteries. Artery 1-5 are the five arteries with no plaque built up; Artery 6-7 are the two arteries with inflammation level of 0.02%; Artery 8 is the artery with inflammation level of 0.8%; artery 9-12 are the arteries with different inflammation levels more than 4%. Data shown as mean ±SD. P < 0.05 compared with control group.

In this research, atherosclerotic plaques were found in seven mouse models. From the immunohistochemistry staining results, we quantified the severity of atherosclerosis by calculating the ratio of CD36(+) area to intima-media area (IMA) as the indicate of inflammation severity, and the ratio of plaque area to IMA as the indicate of anatomical severity. By analysis, we obtained the inflammation severity of 0.043, 0.061, 0.082 and 0.113 in the four arteries, respectively. The anatomical atherosclerosis severity obtained for these arteries were 0.668, 0.528, 0.51 and 0.565, respectively (p < 0.05). In the *in vivo* imaging experiments, the PA signal enhancement of 4.3, 5.2, 8, 16.3 was found from the four arteries (Artery 4-7 in Figure 7). Atherosclerotic plaques with less than 0.008 CD36(+) expression were found in three arteries (Arteries 6-8 in Figure 7 & Supplementary Figure S5). But the PA enhancement of the three arteries was not observed due to the limited imaging sensitivity of the current system. For the other 5 arteries (Arteries 1-5 in Figure 7) from the mouse models, neither atherosclerotic plaque nor PA signal enhancement was found. These results confirm that the enhanced PA signal appeared at the arteries with increased CD36(+) expression. On the other hand, as shown in Figure 7, we did not find a relationship between PA signal and anatomical atherosclerosis severity. The statistic results of PA signal enhancement, CD36(+) expression and plaque area are shown in Figure 7.

## Discussions & Conclusion

The development of atherosclerotic plaque in carotid arteries leads to compromise in blood delivery to the brain, and the rupture of this plaque could cause stroke, leading to high morbidity and motility. As we presented in the introduction, extensive preclinical and clinical studies have shown that the atherosclerotic inflammatory activities are highly related to plaque formation, progression, and ultimate rupture. In this study, we showed the feasibility of a semiconducting polymer NP along with noninvasive PA imaging technology for identifying carotid atherosclerosis inflammation in mouse models and its ability to reflect inflammation severity.

Efficient label of NPs to carotid atherosclerosis inflammatory cells is the prerequisite in obtaining PA molecular imaging of inflammation *in vivo*. Theoretically, appropriate molecular particle size, proper circulation time in blood, and the surface decoration of targeting ligand are three important factors influencing the efficiency of the molecular probes in carotid inflammation labeling 43. In our research, the PBD-CD36 NP's size was around 50 nm, and circulation time in blood was measured to be around 4 h. Our experimental study showed good efficiency of this probe for labeling the inflammatory cell with great specificity. However, for extending the use of molecular probe from experimental study to clinical application, following three features of the probes needs to be studied and confirmed in near future: (1) immunogenicity and toxicity test in big animals is needed, (2) faster and thorough excretability of the probes needs to be confirmed, and (3) red-shift of the absorption wavelength improvement of molecular probes need to be confirmed.

Imaging sensitivity, which mainly depends on molecular probes and PA imaging systems, plays an important role in image quality and detection efficiency. Besides developing high-efficiency molecular probe, improving the excitation efficiency of light is important in improving imaging sensitivity. Especially in *in vivo* imaging, the scattering of light due to biological tissue limits the imaging sensitivity of the target beneath animal skin, thus it is essential for the imaging system to reduce the influence of tissue scattering. In this study, we chose the excitation wavelength at 1064 nm based on the good light absorption of the PBD-CD36 NPs at this wavelength, in the near-infrared range, which has lower scattering in tissues. The PA imaging results in our study demonstrated that 3D information of carotid arteries which locate about 2-3 mm beneath mouse skin is well achievable *in vivo*.

The PA imaging and immunohistochemistry staining were performed on the healthy mouse and the mouse models on HFD. No obvious PA signal enhancement was seen in the healthy mouse. Varied level of PA signal enhancement was observed in mouse models with carotid atherosclerosis plaque built-up (Figure 5). The varied level of signal enhancement is mainly due to different levels of NPs accumulation, which is tightly linked to CD36(+) expression (inflammation levels) in carotid atherosclerosis. The immunohistochemistry staining of the carotid arteries was performed to verify the *in vivo* imaging results. The quantification of the PA signal reflected CD36(+) expression (inflammation severity) with good accuracy, but had little to do with plaque anatomical severity (Figure 7). This result confirms the feasibility of PA imaging in *in vivo* assessing carotid atherosclerosis inflammation, assessing atherosclerosis progression mechanism, and even for predicting atherosclerotic plaque rupture.

In one word, our study clearly showed the feasibility of using PA imaging along with PBD-CD36 NPs to image atherosclerosis* in vivo* and its ability to reflect the levels of inflammation. Since inflammation is a risk factor for plaque rupture, the potential of the system to successively observe the inflammation change with atherosclerosis progression is very critical to investigate the role of inflammation in atherosclerosis generation, progression and plaque rupture. Also, our study established a method to interrogate atherosclerosis and thus determine the intervention if and when needed, benefiting the patient significantly.

One limitation is observed in our research. That is, the relationship between inflammation and plaque rupture risk is not studied. Although amounts of preclinical studies have demonstrated the tight link between inflammation activities and plaque progression and rupture, it is necessary to track the inflammation progression of plaque and its rupture progress using noninvasive PA imaging, thus to define the severity, vulnerability of plaques from PA imaging results. This part is also our next job in near future.

The other limitation is the signal fluctuation. It is notable that PA signal fluctuation exists between the images obtained before NPs injection and 24 h post NPs injection from normal mice in *in vivo* imaging experiments. The PA signal variation makes the PA quantification unreliable in mild inflammation condition. In this study, the mildest inflammation we identified is 4% inflammation level from immunohistochemistry staining results. For the 0.8% inflammation level and less, the PA signal enhancement was immersed into normal signal fluctuation, thus we cannot identify them. But the development of NPs with higher sensitivity and higher labeling efficiency would help. In addition, the PA signal variation was from blood, which has different absorption spectrum from the NPs. Thus the signal variation can be strictly quantified with PA spectrum method. But this method involves multi-wavelengths excitation, pulse energy calibration, etc. The hardware and software of the system need to be improved with complexity, which is what we plan to do in the next step.

In conclusion, we showed that the inflammation level in atherosclerotic plaques in carotid arteries can be assessed *in vivo* using PA imaging modality and NPs targeting. It has many advantages, including no harmful radiation, low cost, high detection specificity, high imaging contrast, good imaging depth resolution, and good compatibility with clinical imaging modalities. The NPs we developed (PBD-CD36) have the potential for noninvasive diagnosis of atherosclerosis, plaque progression research and atherosclerotic plaque medicine treatment, and monitoring of plaque *in vivo* during treatment.

## Supplementary Material

Supplementary figures and information.

## Abbreviations

ALT: Alanine aminotransferase
AS: Atherosclerosis
AST: Aspartate aminotransferase
HFD: High-fat diet
HGB: Hemoglobin
LCA: Left carotid artery
MAP: Maximum amplitude projection
NIR: Near Infrared Region
NP: Nanoparticle
OCT: Optimal cutting temperature
PA: Photoacoustic
PLT: Platelets
RBC: Red blood cells
RCA: Right carotid artery
TCPE: 2, 4, 5-trichlorophenoxyethanol
TEM: Transmission electron microscopy
US: Ultrasound
WBC: White blood cells
