# Supplementary material for: In vivo assessment of inflammation in carotid atherosclerosis by noninvasive photoacoustic imaging
Source: Theranostics. 2020 Mar 26;10(10):4694–704. doi: 10.7150/thno.41211 (PMC7150488; doi:10.7150/thno.41211)
Supplement: Supplementary file 1 — Supplementary figures and information. [file thnov10p4694s1.pdf]

In vivo assessment of inflammation in carotid atherosclerosis by noninvasive photoacoustic imaging

Characterization of PBD and PBD-CD36:

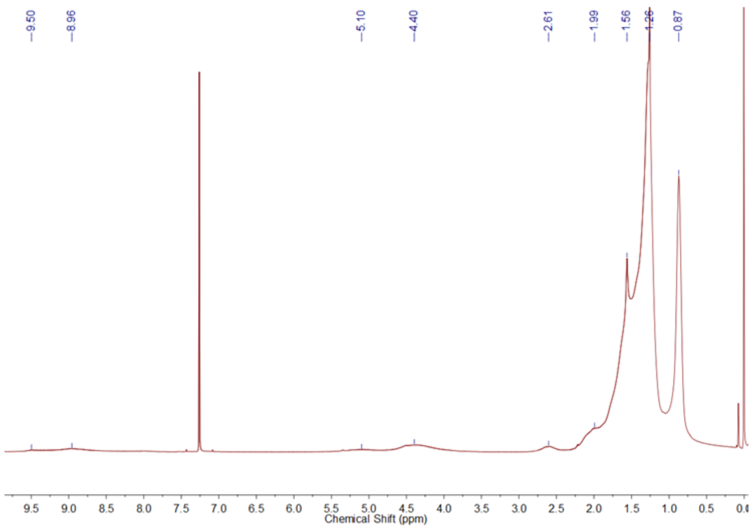

Figure S1. ^{1}H NMR spectrum of PBD in $CDCl_3$.

The Zeta potential of PBD NPs and PBD-CD36 NPs to be −10.7 mV to −20.2 mV, respectively. The obvious decrease in zeta potential demonstrates the successful conjugation of anti-CD36 onto the surface of PBD NPs.

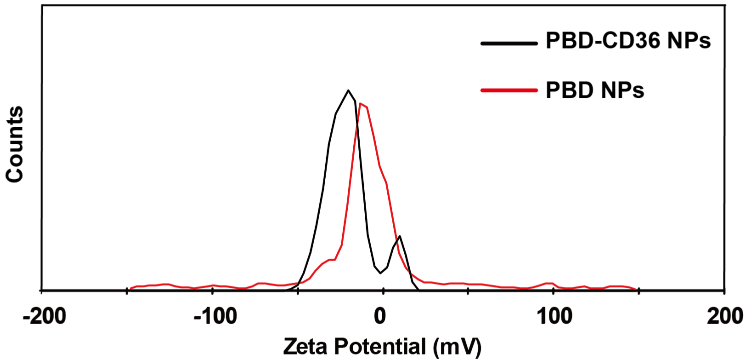

Figure S2. Zeta potential of PBD NPs and PBD-CD36 NPs.

Stability of PBD-CD36:

The stabilities of PBD-CD36 NPs in different media. After incubation PBD-CD36 NPs in FBS, cell culture medium, PBS with different pH for 24 h, the absorbance of PBD-CD36 NPs did not exhibit obvious changes (Figure S3), indicating that PBD-CD36 NPs are stable in various environments.

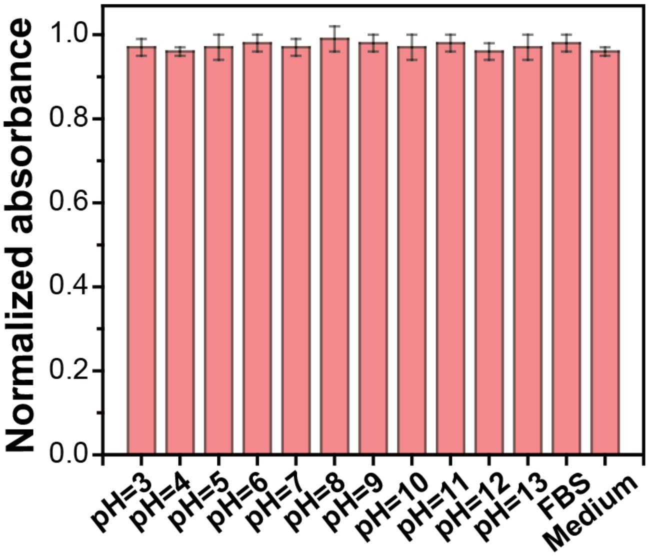

Figure S3. Absorbance of PBD-CD36 NPs in various aqueous media.

The stability of PBD-CD36 NPs in intracellular environment. After the NPs incubated by Raw264 cells, the PA signal of the samples was measured at the time points of 0, 4, 8, 12, 16, 20, 24 h and the stable PA signal showed that the NPs have good stability in intracellular environment.

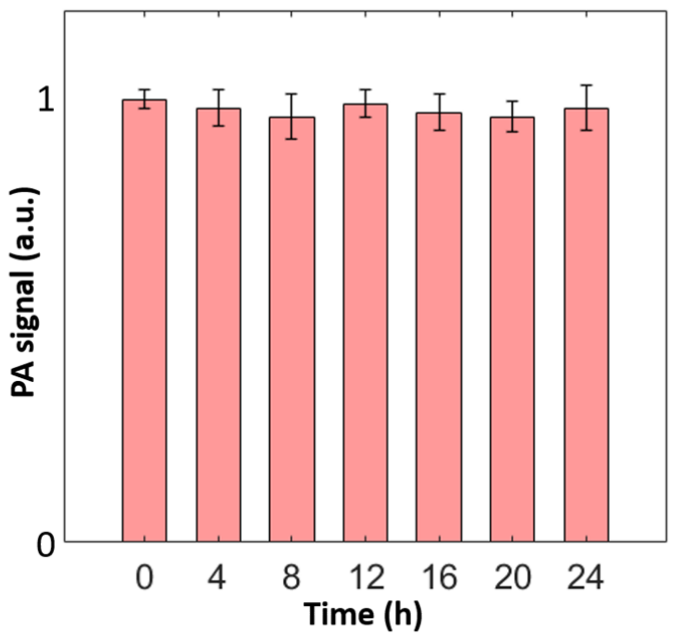

Figure S4. NPs stability in intracellular environment.

Cytotoxicity of PBD and PBD-CD36:

Cytotoxicity experiment at 48 h was performed. In this experiment, the cells were cultured 48 wells in a 96-well plate. We put the PBD and PBD-CD36 NPs with different concentration the wells. The concentration of NPs is 0, 20 μg/mL, 100 μg/mL and 200 μg/mL, respectively. For every concentration and every kind of NPs, we choose the cells in 6 wells to use for future statistic analysis. The experimental results are shown in the figure as below. The result shows good cytotoxicity of the NPs at 48 h.

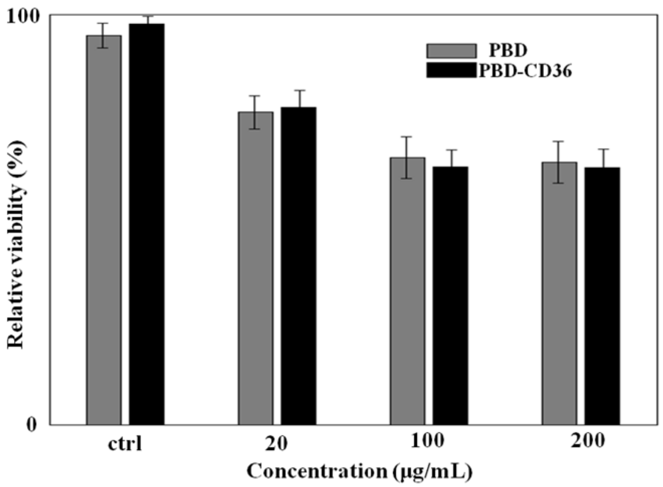

Figure S5. Cytotoxicity of PBD and PBD-CD36 NPs at 48 h.

Cellular uptake:

For in vitro studies, Raw246.7 cells were seeded on two culture dishes (Costar 6 Well Clear Not). After 24 h, the cells were incubated for 4 h with PBD NPs and PBD-CD36 NPs at 37 ^{o}C, respectively and the concentration of PBD and PBD-CD36 NPs was 300 μg/mL. Then the cells were washed with PBS for three times to remove the unconjugated NPs. After digesting the cells from culture dishes, the cells were diluted with PBS to the concentration of 0.125×10^7, 0.25×10^7, 0.5×10^7 and 1×10^7 cells/mL. A PA imaging system was used to observe the PA signal generated with the cell samples.

Fig. S6 shows the PA signal of cells samples with PBD NPs and PBD-CD36 incubated. With the decrease of cell concentration, the PA signal decreased. And the PA signal from the cell samples with PBD-CD36 incubated is obviously higher than that with PBD incubated (Figure S6B). These findings demonstrated that PBD-CD36

NPs could be efficiently internalized by Raw246.7 cells and generate higher PA signals, potentially allowing for the utilization of PBD-CD36 NPs for efficient inflammatory cell labeling and sensitive PA imaging in carotid atherosclerosis inflammation components. .

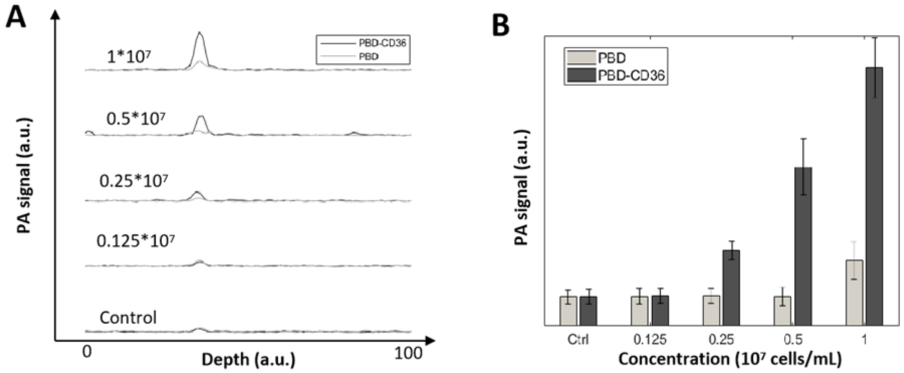

Figure S6. (A) the PA signal of cell samples with PBD and PBD-CD36 NPs incubated, respectively. (B) The quantified PA signal for different samples.

Quantification of PA signal enhancement:

For the photoacoustic images of all arteries, three consecutive B-scan images along the arteries with maximum photoacoustic signal were selected and ROI at the border of photoacoustic signal area in B-scan images was drawn (Fig. S7A-D). To maintain the standardization of photoacoustic signal quantification, same ROI was used in all the selected B-scan images. Photoacoustic signal intensities of every B-scan in these regions were extracted and summed in Matlab software. The ratio of summed intensity post probe injection to that before probe injection of the same artery was considered as quantification of photoacoustic signal enhancement. The quantified photoacoustic signal of five arteries from three mice is shown in Fig. S7E. Note that

photoacoustic signal of mouse 1 LCA is almost the same with mouse 1 RCA, so it is not shown in Fig. S7E.

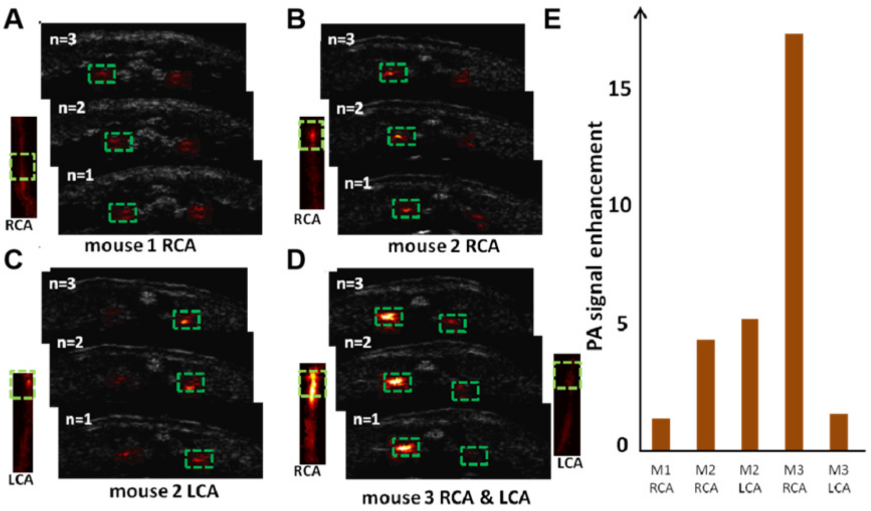

Figure S7. (A-D) are the consecutive photoacoustic-ultrasound B-scan images of the arteries (RCA: right carotid artery; LCA: left carotid artery). Inset images are the MAP photoacoustic images of the corresponding arteries. The green squares on MAP images are the location where the B-scan images were selected. The green squares on B-scan images are ROI (region of interest) for photoacoustic quantification. (E) The quantified photoacoustic signals of five arteries from three mice.

PA signal fluctuation of normal mice:

The PA signal of carotid arteries was performed on five normal mice. For the carotid arteries of every mouse, we choose six ROIs and calculate the PA signal enhancement. The result is shown in Figure S8. The PA signal fluctuated within 18% 24 h post NPs injection and before NPs injection.

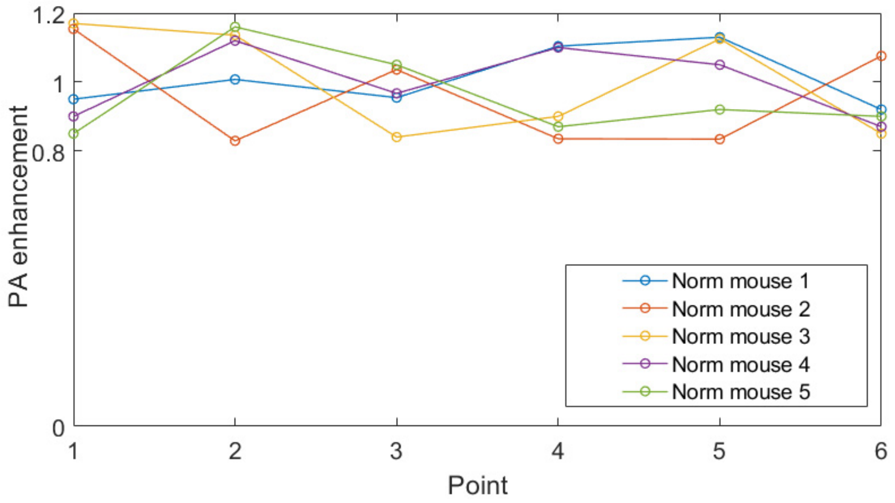

Figure S8. PA enhancement of six region of interest for every mouse. The quantification was based on five normal mice.

CD36 (+) expression and plaque area quantification:

Statistical analysis was performed by Image-Pro Plus software. CD36-possitive areas and atherosclerotic plaque areas were calculated. The plaque area and CD36 (+) expression area are marked and calculated by Image-Pro Plus software as shown in Figure S9. Differences were considered significant at p-value < 0.05.

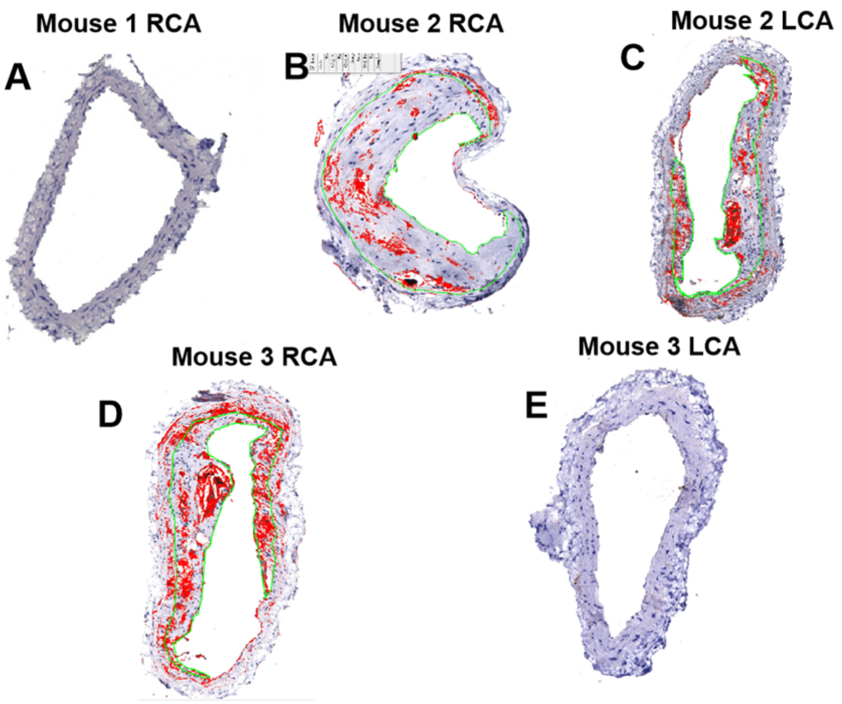

Figure S9. Plaque area and CD36(+) expression area marked and calculated by the software of Image-Pro Plus.

Immunohistochemistry staining of carotid arteries from atherosclerotic mouse models

In four carotid arteries with atherosclerotic plaques successfully built, the CD36(+) expression is 8%, 0.75%, 0.06% and 0.062%, respectively (Fig.S10). We found obvious PA enhancement in the carotid artery with the 12% CD36 expression (Fig.S10(a)). But no PA signal enhancement was found in *in vivo* PA imaging 24 h post probe injection for the arteries (Fig.S10(b), (c) and (d)). It means that the current system is not sensitive enough to identify such mild inflammation level.

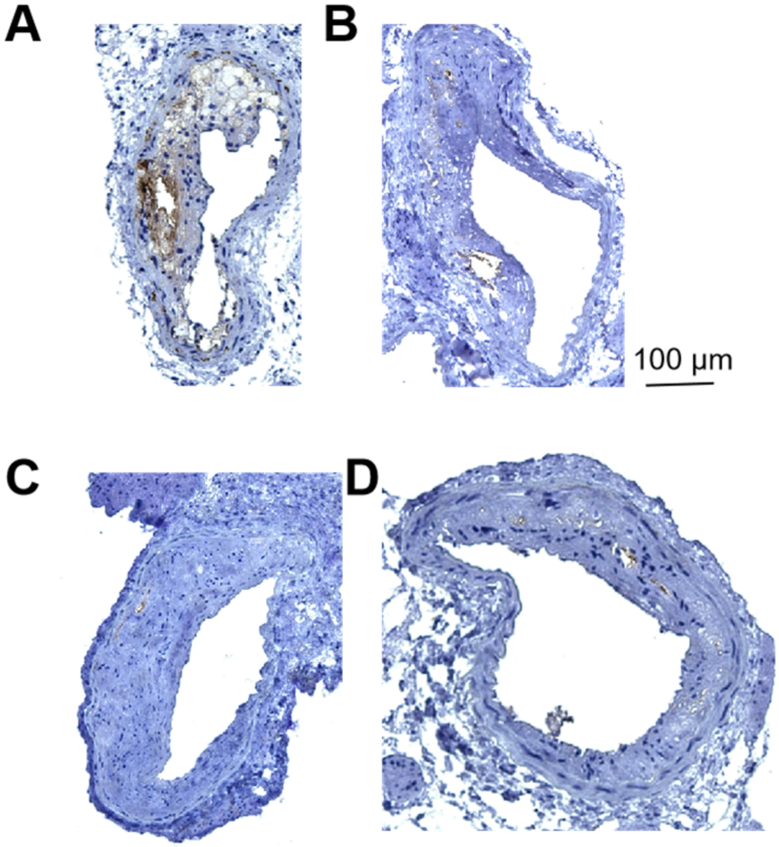

Figure S10. (A-D) The results of anti-CD36 staining of the atherosclerotic plaques. CD36(+) expression is 8%, 0.75%, 0.06% and 0.062%, respectively.

In five arteries from mouse models, no atherosclerotic plaque was found and almost no CD36 expression in these arteries. Correspondingly, we did not find PA enhancement from the *in vivo* imaging results. Four staining images from the five arteries are shown in Figure S11.

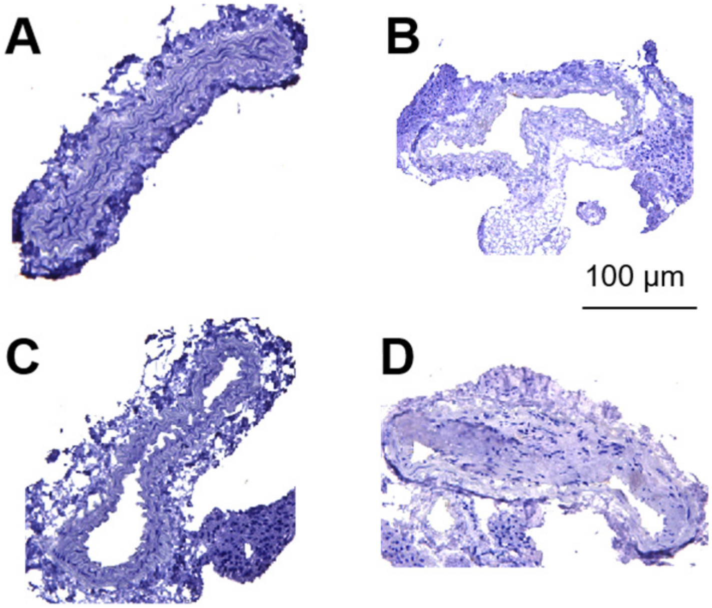

Figure S11. Anti-CD36 staining of the carotid arteries with no plaque found.
